# Evaluation of a home-based transcranial direct current stimulation (tDCS) treatment device for chronic pain: study protocol for a randomised controlled trial

**DOI:** 10.1186/s13063-015-0710-5

**Published:** 2015-04-23

**Authors:** Francis O’Neill, Paul Sacco, Turo Nurmikko

**Affiliations:** Pain Research Institute, Clinical Sciences Centre, Lower Lane, Fazackerley, Liverpool, L9 7AL UK; Department of Oral Surgery, Liverpool University Dental Hospital, Pembroke Place, Liverpool, L3 5PS UK

**Keywords:** Pain, Brain stimulation, Motor cortex (M1), Transcranial direct current stimulation (tDCS), Repetitive transcranial magnetic stimulation (rTMS)

## Abstract

**Background:**

Stimulation of the primary motor cortex (M1) has been shown to reduce the pain of neuropathy in multiple studies. There are several methods of stimulation both invasive and non-invasive. Recent work by this laboratory has seen that 40% of a sample of chronic neuropathic pain patients responded positively to non-invasive repetitive transcranial magnetic stimulation (rTMS) to the motor cortex with a reduction in pain levels by at least 20%. The effect however is short lived and multiple return visits are necessary to maintain this response. Transcranial direct current stimulation (tDCS) offers a more mobile method of motor cortex stimulation and is similarly non-invasive. The protocol described is designed to assess the analgesic effect of a home-based tDCS treatment device on chronic neuropathic pain in both responders and non-responders to previous TMS treatment.

**Methods/design:**

This article reports the protocol for a randomised, sham-controlled, double-blinded crossover study in which patients with chronic neuropathic pain (*n* = 24) will receive anodal, cathodal and sham tDCS over M1. All patients will have previously completed a study of rTMS of the motor cortex and have been designated as responders or non-responders to this modality.

Patients receive all three tDCS stimulation types by self-administration. We assess the effect on pain scores [numerical rating scale (NRS)], self reported health status (Short Form-36 Health Survey) and anxiety/depression (Hospital Anxiety and Depression Scale). A linear mixed model with fixed effects will analyse changes in pain scores from pre- to post- interventions. Analysis will be carried out on an intention-to-treat basis. A proportion analysis will also be carried out with patients separated into either responders or non-responders to previous TMS.

Safety will be assessed throughout the study by monitoring of adverse events.

**Discussion:**

The result of this trial will assess the efficacy of self-administered tDCS of the motor cortex in the treatment of chronic neuropathic pain and also provide insight into whether a potential differential effect is seen in patients that have previously been shown to be either responsive or non-responsive to rTMS over the same area.

**Trial registration:**

ISRCTN56839387 date 27 January 2014. First patient randomised to trial 30 October 2012.

## Background

Stimulation of the primary motor cortex (M1) of the brain was first shown to reduce central pain in patients by Tsubokawa et al. [1] using implanted electrodes in the epidural space around the brain. More recently, non-invasive methods of cortical stimulation have been tested such as repetitive transcranial magnetic stimulation (rTMS) and transcranial direct current stimulation (tDCS) [2].

Previous research in our laboratory has shown that approximately 40% of chronic pain patients obtained at least 20% improvement in pain scores following high-frequency rTMS of the M1 motor cortex [3] lasting for approximately 2 weeks consistent with other rTMS studies [4,5]. The effect seems to be maintained as long as rTMS sessions continue, but this requires frequent attendances by patients. The proposed study is based on the hope that tDCS could offer an alternative method for cortical stimulation in this group of patients. As portable tDCS devices have become available, patients/carers can be trained in its application and patients may use these at home without the need for regular visits to our laboratory. If proven sufficiently effective, tDCS could offer an economical and convenient way of delivering cortical stimulation for pain relief.

tDCS involves the application of a low-amplitude constant electric current to the scalp, a proportion of which enters the skull to affect cortical activity in the brain. It does not directly induce action potentials in neurons but rather alters the membrane potential and thus influences the excitability of these cells [6,7]. Typically it is applied via two electrodes, an anode and a cathode, with the current travelling from anode to cathode. The polarity however can be changed by reversing the electrode position. Anodal stimulation of the scalp overlying the cortex results in increased excitability of this cortical area whilst cathodal stimulation has the opposite effect [8].

It has been shown to be effective in the treatment of various conditions including motor function recovery following stroke, reduced epileptiform discharges in epilepsy, mood elevation in depression and improvement of motor function in Parkinson’s disease [9-12].

Several studies have also shown it reduces the perception of acute pain [13,14] and experimentally induced acute pain in healthy volunteers [15,16] and in post-operative pain [17]. In chronic pain conditions that are refractory to medical treatment, anodal tDCS stimulation has been shown to reduce pain (at least in the short term) in fibromyalgia [18,19], pelvic pain [20], cancer pain [21] and spinal cord injury [22,23], although in spinal cord injury pain recent studies have not replicated positive results [24]. In a crossover trial where volunteers were exposed to both anodal and sham tDCS over the M1 motor cortex hand area, Antal et al. [25] demonstrated a reduction in chronic pain scores and a normalisation of intra-cortical inhibition. These effects persist for several weeks after treatment and may reflect long-term plastic changes at neuronal synapses [26-29].

tDCS of the motor cortex also influences the activity of areas distant to the site of stimulation and this is probably achieved through inter-neuronal connections between these sites [30]. This is supported by imaging studies in humans, indicating that cortical stimulation reduces pain by modulating activity in networks of brain areas involved in pain processing, such as the thalamus or by facilitating descending pain inhibitory mechanisms [31-34]. Side effects are uncommon and relatively minor (mostly headache, itch and skin redness) and it is considered a very safe form of treatment with a few contra-indications [35-37].

### Aims

The main aim of this study will be to determine the efficacy of home-based tDCS treatment in two chronic neuropathic pain patient populations: responders and non-responders to rTMS. The hypothesis is that those patients who have previously responded to rTMS will also respond to tDCS while non-responders may not. To test this hypothesis TMS responders will be subjected to three different types of tDCS: anodal, cathodal and sham tDCS. In addition, there remains a possibility that tDCS may show efficacy even in those patients who previously have not responded to rTMS. Therefore, non-responders will be subjected to the same three modes of stimulation as responders.

## Methods/Design

Ethical approval for this study was granted from the National Research Ethics Committee REC reference 12/EE/0315.

### Patient selection

Patients will be selected based on response to previous treatment with rTMS for chronic pain, i.e., all subjects, both responders and non-responders from previous rTMS studies, will be invited to participate. All subjects shall be informed of the purpose of the experiment and written consent to enter the study will be obtained prior to entering the study by the principal investigator.

#### Inclusion criteria

Inclusion criteria include (1) age between 18–85 years; (2) stable analgesic medication for the prior month; (3) mean pain levels ≥4 out 10 on VAS for pain during the 1-week run-in phase, based on patient diary; (4) willingness to take part; (5) ability to consent; (6) previously had a minimum of five sessions of TMS for pain, and can be named as a “responder” or “non-responder” where the responder reports a weekly average pain reduction of 20% on an NRS of 0–10, following five rTMS sessions.

#### Exclusion criteria

Exclusion criteria are (1) severe pain of other origin, e.g. musculoskeletal pain that in the opinion of the investigator may interfere with the reporting of the neuropathic pain being targeted; (2) metal implants/coils/electronic devices; (3) drug or alcohol abuse; (4) pregnancy; (5) psychiatric or psychological disorders; (6) epilepsy; (7) inability to understand instructions or operate equipment; (8) high-dose opioids; (9) uncontrolled medical conditions (e.g. active cancer, uncontrolled renal, pulmonary or cardiac disease). (As the patients recruited have passed the stringent rTMS study entry criteria, these restrictions are unlikely to prevent many entering.) Patients were diagnosed with neuropathic pain, according to the new classification [38]. Two specialists (neurologist, pain specialist) had to agree on the diagnosis. Alcohol and drug abuse was ruled out during the interview and review of medical records.

### Study design

This study will be a randomised, double-blind, double cross-over, sham-controlled trial. Three stimulation modes will be compared: anodal, cathodal and sham stimulation.

Twelve responders and 12 non-responders to rTMS will be randomly assigned to receive anodal, cathodal or sham stimulation as their first treatment session.

### Randomisation

Randomisation will be done by a research clerk not otherwise involved in the study. Computer-generated numbers will be used to create the participant numbers and order lists. The latter will be placed in a numbered opaque envelope and sealed. The copy of the order lists and participant numbers is kept by the research clerk.

#### Initial and follow-up assessments

The initial and follow-up assessments will include a record of (1) handedness (not in follow-up assessments); (2) NRS for pain, including measurements for overall daily pain severity, unpleasantness of pain and severity of paroxysmal pain attacks if present. Pain diaries will be filled out daily for 1 week at baseline and for 14 days beginning at each stimulation period [39]. (3) Health status will be assessed by the Short Form 36 Health Survey (SF-36) [39]. (4) Anxiety and depression will be assessed by the hospital anxiety and depression scale (HADS) [40], as well as (5) the mini-mental state examination [41] and (6) patient experience of equipment use, including convenience and adverse effects [39]. Patients are given contact details of medically qualified personnel to contact in case of serious side effects. These will be reported descriptively.

### Determination of the stimulation site

The stimulation site will be the same as used during previous TMS treatments. In practice, this will mean placing the anodal/cathodal electrode over the primary motor or premotor cortex contralateral to the pain. All patients have previously had an MRI of the brain and their localisation on the scalp of the previous TMS treatment can be easily obtained using the brain navigation system associated with the PRI TMS device (eXimia, Nextsim, Helsinki, Finland). In the few cases where this is not possible, the location will be based on bony landmarks as advised by Fregni et al. [10]. In those cases where multiple sites were stimulated using TMS, the site of best response will be chosen.

Direct current will be delivered from a battery-driven, constant current stimulator (Magstim HDCStim stimulator, The Magstim Co., Whitland, UK) using saline-soaked surface sponge electrodes (5 × 5 cm) placed over the previously identified area to target the motor cortex (M1) and over the contralateral supraorbital area. The device will be worn for 20 min each day for 5 days consecutively beginning on the first day of their visit. During active treatment the current delivered will be 1.4 mA with a size 25 cm^2^ electrode. (Changing the direction of the current will result in either anodal or cathodal stimulation with the M1 stimulation being the determinant of which mode is being used.) All subjects are asked to remain non-active during this time and engage in either light reading/watching television or listening to the radio.

### Sham intervention

For sham stimulation, the electrodes are placed in the same positions as for anodal M1 stimulation; however, a constant current of 1.4 mA is only delivered for 30 s (10-s ramp on). During active tDCS treatment, subjects typically report tingling sensations under the electrodes, which rapidly fade [37]. Our sham intervention is therefore designed to provide an initial period of tingling so similar sensations are perceived during active and sham tDCS protocols. This sham protocol has been used by previous investigators [22,42]. Data and instructions on the device display are identical in active and sham settings. Patients will be asked if they can guess the order of stimulation or if they identify any differences in stimulation sessions. This guess will be checked against the actual stimulation order after unblinding.

A research assistant (not otherwise connected with the study) will set the stimulation characteristics as indicated by the information in a sealed envelope opened at the patient visit (anodal/cathodal/sham). This will be repeated at the two subsequent visits. The mode of stimulation is not revealed to the patient or investigators.

### Patient-administered stimulation

At the first visit subjects are measured up for individually fitted headband locators for electrode placement. Headbands are measured and marked relative to ear tragi, the nose and midline of scalp. A separate occipital band ensures the locator headband cannot slip from its position. Anodal and cathodal electrodes are differently coloured (red and black) and the patient is shown which way around they should be placed. Once fitted, subjects are shown how to position the headbands reproducibly and instructed on how to position the band markers relative to the anatomical landmarks described. Patients are then asked to demonstrate this to the investigator and feedback given until electrodes are correctly and reproducibly positioned. Photographs and measurements are taken in case of the need for reference at home and all instructions are given to the patient in writing also. In this patient information leaflet subjects are informed of potential side effects such as tingling, burning sensation, dizziness, headache, nausea and phosphenes while switching the device on or off abruptly. Electrode placement is re-checked at the beginning of each treatment block.

Programming of devices requires a dedicated programmer with security code access. Overriding or manual reprogramming is not available to patients.

### Study flow

The study will have five phases. At baseline (visit 0, one of their rTMS treatment visits), the patient is given a short presentation of how the tDCS device is used with one of the investigators being the substitute patient. The patient will then receive the Patient Information Sheet and Pain diary. If they wish to join the study they will contact the Research Clerk after having read the information after a minimum of 24 h. The Research Clerk will provide the appointment for visit 1, advising the patient to fill in the pain diary over a minimum of 7 days. At visit 1, eligibility will be determined (as per inclusion and exclusion criteria). Provided they are eligible patients will be recruited and consented. The patient and carer/partner will then be shown how to apply the tDCS device, and the patient will then try it on themselves, with the help of the carer/partner if necessary. The first trial stimulation will be carried out according to the randomisation order by the research assistant. The investigators will then confirm that the patient and carer/partner have understood all details of the stimulation. Following instructions regarding the daily use of the device, a further pain diary is supplied and a subsequent visit agreed in 2 weeks’ time. At this visit (visit 2), the patient will return the pain diary and will surrender the tDCS device. The third visit (3) will be arranged for a further 4 weeks’ time (wash-out period); during the interim a daily pain diary will be kept. At visit 3, the tDCS device will be returned to the patient, and a new stimulation mode will be selected by the research assistant as per the randomisation order. After 2 weeks (visit 4), the tDCS device will again be surrendered to the investigators and the second wash-out period of 4 weeks with diary keeping started. At visits 5 and 6, tDCS device will be received and later surrendered by the patient as during previous treatment arms. The final visit (7) will happen in a further 2 week’s time.

In the case of patients who wish to continue, the tDCS device will be given him/her for regular daily use and visits agreed at 1 month, 3 months, 6 months and 12 months. Before each visit, they are asked to keep a 1-week pain diary to assess their mean pain level.

### Evaluation of tDCS delivery

#### Primary and secondary outcome measures

The primary outcome measure will be the mean daily pain intensity during each mode of tDCS. Secondary outcome measures will be change in hospital anxiety and depression scale (HADS), mini mental state examination and the general health survey (SF 36). Monitoring of device usage will also be recorded, along with patient experience of using the device. At the end of each treatment the device will be returned and interrogated to record actual date, time, duration and number of stimulations delivered and if stimulations were successful.

### Sample size calculations

As this is a pilot study, it is based on an opportune sample of 24 patients who have previously undergone rTMS and expressed willingness to take part in the study. No formal power calculations have therefore been carried out. If, however, 8/12 responders receiving anodal tDCS report a beneficial effect [weekly mean reduction of 30% or 2 units on NRS (0–10) from tDCS] and just 2/12 of non-responders do the same, this itself would give a statistical significance of *p* = 0.036 (two-tailed Fisher’s exact test). Other results would yield important data for future studies (see below).

### Statistical analysis

A linear mixed model will be formed with fixed effects of mode of tDCS, treatment sequence, responder group and treatment period, and the random effect as patient (nested in sequence); the model will be first fitted with the pain intensity data. All patients will be included and all pairwise comparisons will be made with the estimated contrast from this model. Secondary continuous outcome measures will be analysed in the same way as the primary endpoint. Baseline covariates will be incorporated as standard fixed effects but the sensitivity of the results to other ways of incorporating baseline information will be assessed [43]. This information will be used to inform future studies. Where required, change in pain scores pre-post each treatment period will be calculated as the difference between pain during run-in baseline and pain over 3 consecutive weeks following tDCS treatment. Analysis will be performed using Stata software (StataCorp, *Stata Statistical Software*, College Station, TX: StataCorp LP). A proportion analysis will also be carried out with patients separated into responders and non-responders and their response rated as positive (pain reduction from run-in baseline of at least 2 units on VAS 0–10, or 30%) and negative (pain relief less) and analysis carried out separately for the three different tDCS treatments.

## Discussion

With the effectiveness of non-invasive M1 stimulation recently demonstrated there has been a call from patients to facilitate more convenient forms of this therapy. It has been in direct response to this patient lead call that the present exploration of home-based non-invasive tDCS of M1 has been devised. Self-administration of tDCS obviously has both advantages and disadvantages. While it may not confer the same accuracy and consistency as clinic-based, professionally administered stimulation sessions, it does reflect the necessity for patients to be able to administer this type of therapy in the convenience of their own home. It is recognised that there will be limitations on the ability to control timing of stimulus applications and there may be some variability in technique. However, all subjects will be thoroughly briefed in the correct positioning of electrode placement and measurements will be made against previously taken MRI scans and navigated TMS data. These measurements are transferred on to securely locating headbands that are fitted individually to each patient. Each subject is observed while placing the electrodes on a number of occasions to ensure correct technique. The study described is very much a real-world test of both the effectiveness and practicality of using tDCS in this patient population, with a method of delivery that is based on other portable home-use devices such as transcutaneous electrical nerve stimulation, which has been in use for several years, albeit with varying success. If a positive effect is demonstrated it may represent an important cost-efficient option for non-invasive motor cortex stimulation in the treatment of chronic pain.

## Trial status

This trial is on-going and actively enrolling.

## Abbreviations

tDCS: Transcranial direct current stimulation
rTMS: Repetitive transcranial magnetic stimulation
NRS: Numerical rating scale
